# A Rare Case of Septic Knee Arthritis Caused by Clostridium perfringens in a Patient With Colostomy

**DOI:** 10.7759/cureus.16823

**Published:** 2021-08-02

**Authors:** Mohammad U Khubaib, Brett E Stark, Benjamin Gross, Michael L Gross, Oscar Vazquez

**Affiliations:** 1 Sports Medicine, Active Orthopedics & Sports Medicine, Hackensack, USA; 2 Orthopaedic Surgery, Active Orthopedics & Sports Medicine, Hackensack, USA

**Keywords:** sports medicine, orthopedics, internal medicine, infectious disease, sepsis, septic arthritis, knee arthroplasty, synovectomy, clostridium perfringens, colostomy

## Abstract

Septic arthritis of the knee is commonly caused by aerobic organisms. Rarely, it can be caused by *Clostridium perfringens*, usually due to penetrating trauma. This is a rare case of *C. perfringens* septic arthritis in a patient with colostomy due to hematogenous spread. The patient was treated successfully with a synovectomy and a prolonged intravenous antibiotic course. The case report summarizes the existing literature on the topic and discusses the diagnosis, management, and prognosis of such cases as well.

## Introduction

Septic arthritis is an infection of the synovium in any joint of the body. Acute monoarticular arthritis, especially that of the knee, is most often caused by aerobic organisms, with only approximately one percent accounted for by anaerobic species [1,2]. Current literature documents very few cases of *Clostridium perfringens* septic arthritis, and the ones that exist are predominantly secondary to penetrating trauma to the joint [3]. Other presentations have been described in immunocompromised patients, presumably caused by hematogenous spread from the gastrointestinal tract [3]. It has also been reported after anterior cruciate ligament repair surgery [4]. We present a rare case of *C. perfringens* septic arthritis in an immunocompetent patient by hematogenous spread. We also summarize the patient’s presentation and successful treatment and review the literature as well.

## Case presentation

This is the case of a 98-year-old female with a long history of severe end-stage osteoarthritis, for which a total knee arthroplasty had been recommended for many years but the patient refused any kind of surgery due to her advanced age. She was treated with nonsteroidal anti-inflammatory drugs (NSAIDs), bracing, and physical therapy with bi-annual cortisone and hyaluronic acid injections to control discomfort. She was also treated at the practice intermittently for a large popliteal cyst with recurrent effusions via aspiration. The patient ambulated mostly without assistive devices, occasionally using a cane, and was high functioning in her activities of daily living. She lived at home with her daughter. Her past medical history was significant for multiple joint osteoarthritis, hypercholesterolemia, allergic rhinitis, and a remote history of large intestine adenocarcinoma in remission for over 10 years, successfully treated with resection and maintained colostomy. 

The patient presented to the office complaining of several days of increasing knee pain. Her chief complaint was pain and swelling in the posterior knee although she was able to ambulate and bend her knee. On exam, she had a large popliteal cyst. She had mild swelling of the knee, no discernable effusion, no redness, no warmth, and intact skin. Her range of motion was 5-130 degrees, which was typical for her. She denied trauma, fevers, chills, or other constitutional symptoms. She was treated with aspiration of the cyst and cortisone injection in the joint and was discharged with a plan to inject hyaluronic acid in two weeks.

**Figure 1:**
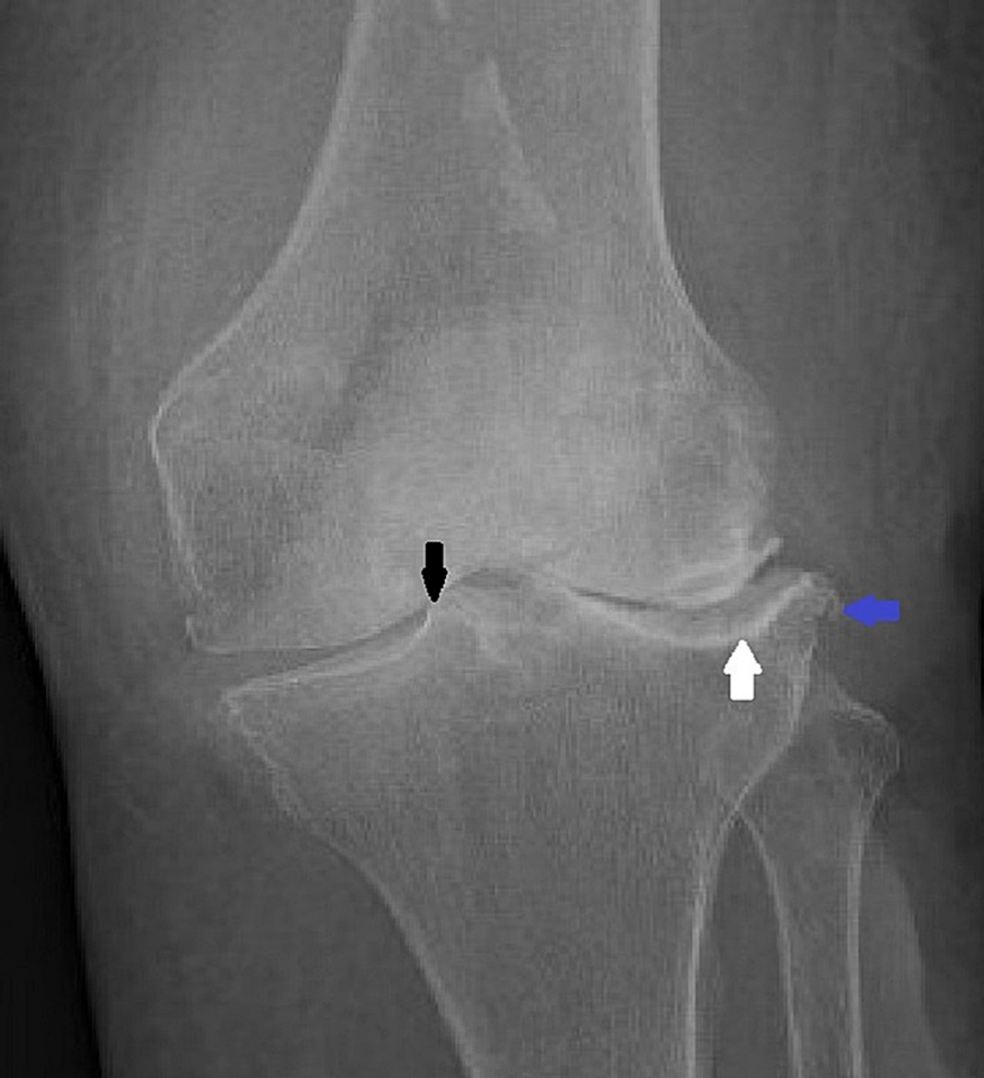
Plain radiograph of the knee showing severe osteoarthritis. Black arrow showing extensive loss of cartilage and "bone-on-bone" disease, white arrow showing bony sclerosis, and blue arrow showing osteophyte.

The next morning, the patient presented to the emergency room. She stated that she had experienced marked improvement in her symptoms following treatment in the office, however, awoke the next morning with a hot, erythematous, swollen knee with limited range of motion or ability to ambulate. On exam, the patient was afebrile (temperature: 98.3°F) and stable (blood pressure: 147/76 mmHg, HR 60 beats per minute, oxygen saturation on room air >96%). Her knee was aspirated in the emergency room and found to have elevated WBCs (87,000 cells/mm³) with 98% neutrophils and a positive Gram stain. Her serologies showed leukocytosis to 24.7 cells/mm³, elevated erythrocyte sedimentation rate (ESR) of 37 mm/hr, and C-reactive protein (CRP) of 2.1 mg/L. Plain radiographs of her left knee showed end-stage osteoarthritis consistent with prior imaging without evidence of new trauma or osteomyelitis (Figure 1). The patient was then taken to the operating room for arthroscopic irrigation, debridement, and synovectomy. Purulent thick synovial fluid was encountered and sent for analysis. There was severe end-stage tri compartmental arthritis, most severely patellofemoral and lateral, with little remaining joint cartilage. This pre-existing degenerative disease limited the ability to assess infectious cartilage damage. There was also extensive edematous, hyperemic, and boggy tri compartmental synovitis. Care was taken to remove as much of the synovium as possible, and a drain was left in the joint to allow for effective postoperative drainage. The patient was started on broad-spectrum antibiotics (vancomycin and ceftazidime) and probiotics at this time per infectious disease consultant recommendations.

The cultures of the initial aspiration and washout grew *C. perfringens*. The patient denied recent trauma to the knee and denied recent possible outdoor exposures due to Covid-19 pandemic restrictions. She did, however, have a history of colon cancer treated greater than 10 years prior. Colon adenocarcinoma has previously been found as a nidus for the hematogenous spread of atypical bacteria such a *C. perfringens* to joints [5]. The patient was status post resection with a permanent colostomy and adjuvant chemotherapy with resultant eradication of the disease. She did not have active disease at this time to her or her family’s knowledge. The diagnosis of *C. perfringens* septic arthritis prompted a full work-up with a CT scan of her chest/abdomen/pelvis and a PET scan. No recurrence of the adenocarcinoma was found.

The cultures demonstrated penicillin sensitivity, prompting a switch from broad-spectrum antibiotics to IV penicillin. The patient’s pain improved significantly (9/10 to 3/10) after the arthroscopic irrigation and debridement, and she required up to two doses of opioids daily for analgesia. Her swelling, redness, and pain also improved drastically, with an associated increase in range of motion from minimal to 5-60 degrees. The patient was however slow to bear weight on the knee, with physical therapy started on the second postoperative day. Aspiration postoperative day four returned minimal fluid. The patient received appropriate deep vein thrombosis prophylaxis throughout her hospital course. 

**Figure 2:**
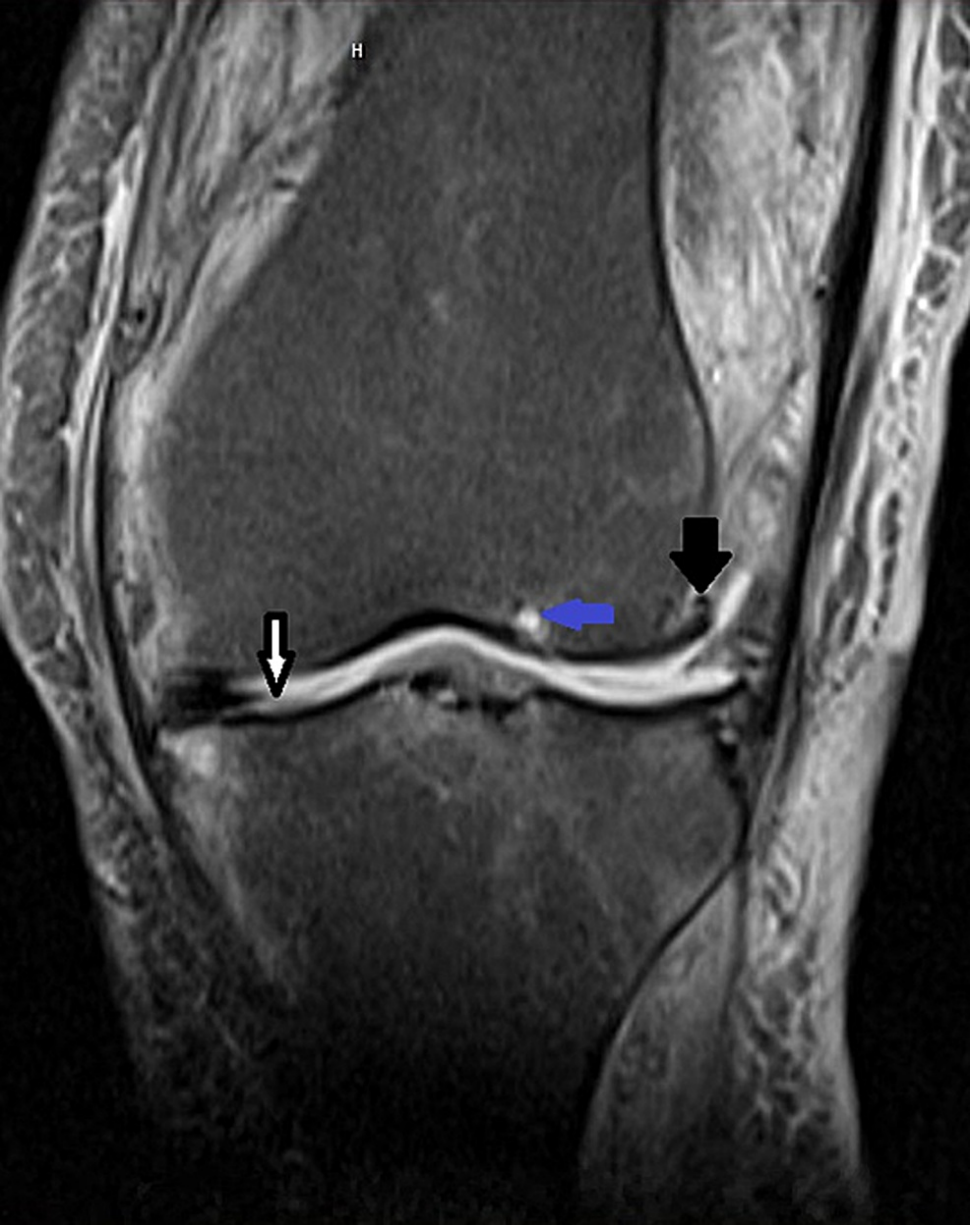
MRI of the knee showing severe osteoarthritis. White arrow showing extensive loss of cartilage, blue arrow showing subchondral cyst, and black arrow showing osteophyte.

The patient was discharged to rehabilitation, where she spent one month recovering. In week six of treatment, she developed an allergic reaction to penicillin (rash) and was readmitted to the hospital with a medication change to clindamycin. An aspiration of her knee at that time did not return fluid. Interventional radiology then performed a guided aspiration with a minimal fluid return. Cultures of this fluid were negative. As the patient’s leg was still swollen and her weight-bearing difficulty remained, an MRI was ordered to evaluate for osteomyelitis or the presence of abscess or soft tissue involvement. The MRI showed only a mild effusion and severe arthritis of the joint (Figure 2). Doppler ultrasound was also negative for DVT. Antibiotics were discontinued at this time as there was no sign of persistent infection with negative serologies and culture. Over the following week, the patient’s leg swelling and ability to ambulate improved with physical therapy, and her residual swelling was attributed to post-infectious sequelae in the setting of severe osteoarthritis. The patient returned to rehabilitation and continued to improve until discharge to home. Throughout her course, the patient did not develop fevers, chills, or any other constitutional symptoms.

## Discussion

Septic arthritis is a rare, but serious, medical emergency with known incidence rates between 4-29/100,000 patient-years and increasing due to a variety of iatrogenic and population-based factors [6-8]. It can be effectively caused by any organism and has multiple etiologies, such as hematogenous spread through the well-vascularized synovium or direct joint introduction via environmental or nosocomial trauma or puncture that otherwise exposes the synovium to infecting pathogens [9]. Hallmark symptomatology includes a red, warm, swollen, and painful joint with a limited range of motion; however, a presentation may vary irrespective of the offending agent [1]. Patients may have signs of bacteremia such as fevers and chills, but, like this case, these may not occur. Degeneration of the synovium and articular cartilage also may or may not occur. The differential diagnosis for a patient with this presentation includes inflammatory etiologies such as rheumatoid arthropathies and crystallopathies, degenerative pathologies such as osteoarthritis, trauma, or infectious insults. 

While largely due to aerobic organisms, approximately one percent of septic arthritis cases are caused by anaerobic infections such as clostridial species [2]. Clostridia are Gram-positive, spore-forming, saprophytic, and obligate anaerobic bacilli [10]. They can be found ubiquitously, including in the human gastrointestinal tract [10]. A literature review from 1966 to 2014 found 55 cases of clostridial septic arthritis, of which *C. perfringens* was the most commonly involved species [11]. *C. perfringens* septic arthritis is predominantly associated with direct introduction to the joint space via contamination of a traumatic instrument or the overlying skin, as well as hematogenous spread in immunocompromised individuals [12,13]. A direct introduction may have been the infectious process in this case as our patient’s colostomy increases the likelihood of accidental soiling of the overlying skin by gastrointestinal flora. When the patient’s baker’s cyst was therapeutically aspirated or when the joint was injected with steroids, despite appropriate aseptic precautions, the extremely hardy spores could have prevailed, introducing *C. perfringens* to her joint through the needle track. While this is a possibility, the timeline more likely suggests a hematogenous spread. If contamination of the joint occurred during the office procedures, symptoms would have been expected to manifest to septic arthritis in the following days, not approximately after 12 hours as seen in this case. The patient may have already had an early infection when she presented to the office. Ruling out recurrence of colonic adenocarcinoma and immunocompromised state, the bacteria could have entered her bloodstream through a microtear in her intestinal lining, irritation at her stoma, etc. Given the rarity of *C. perfringens* septic arthritis, the timeline from inoculation to significant symptoms is not well described or well known, and so our patient’s true infectious nidus remains speculative. Last but not least, septic arthritis being the manifestation of underlying endocarditis should also be considered, but the lack of any constitutional or cardiovascular symptoms, combined with her extremely old age (98) and refusal of invasive procedures, prevented cardiac exploration in this case.

Proper surveillance of *C. perfringens* infections is vital as the endotoxin produced by the bacteria acts as a lecithinase and can lead to gas gangrene. While not commonly seen in association with joint infections, contiguous myonecrosis has been reported [14]. Associated osteomyelitis requiring joint resection was reported from another clostridial species [15]. Given the enhancement of the inherently high acuity in septic arthritis, it is important to consider clostridial infection in cases associated with trauma or when risk factors favor spread from the gastrointestinal tract, such as intestinal carcinoma, diarrheal illness, or colostomies. Once suspected, anaerobic cultures should be obtained along with the aerobic cultures. Standard treatment usually consists of synovectomy and IV penicillin for six weeks [12]. With timely diagnosis and treatment, most patients have good functional outcomes, as was seen in our patient.

## Conclusions

Septic arthritis is a serious complication of joint trauma, therapeutic procedures, or more rarely, hematogenous spread. Although rare, cases can be caused by *C. perfringens*. This diagnosis should be considered when there is a history of trauma, immunosuppression, and/or risk factors for spread from the gastrointestinal tract. Prompt treatment with synovectomy and IV penicillin for six weeks results in desirable functional outcomes.

## References

[1] Shirtliff ME, Mader JT (2002). Acute septic arthritis. Clin Microbiol Rev.

[2] Lorber B (1979). Gas gangrene and other clostridium-associated diseases. Mandell, Douglas, and Bennett's principles and practice of infectious diseases.

[3] Gredlein CM, Silverman ML, Downey MS (2000). Polymicrobial septic arthritis due to clostridium species: case report and review. Clin Infect Dis.

[4] Farooq AH, Dabke HV, Majeed MA, Carbarns NJ, Mackie IG (2007). Clostridial wound infection following reconstruction of the anterior cruciate ligament using bone-patella-bone autograft. J Coll Physicians Surg Pak.

[5] Chodos MD, Johnson CA (2009). Hematogenous infection of a total knee arthroplasty with Klebsiella pneumoniae in association with occult adenocarcinoma of the cecum. J Arthroplasty.

[6] Kennedy N, Chambers ST, Nolan I, Gallagher K, Werno A, Browne M, Stamp LK (2015). Native joint septic arthritis: Epidemiology, clinical features, and microbiological causes in a New Zealand population. J Rheumatol.

[7] Mathews CJ, Weston VC, Jones A, Field M, Coakley G (2010). Bacterial septic arthritis in adults. Lancet.

[8] McBride S, Mowbray J, Caughey W (2020). Epidemiology, management, and outcomes of large and small native joint septic arthritis in adults. Clin Infect Dis.

[9] Klein RS (1988). Joint infection, with consideration of underlying disease and sources of bacteremia in hematogenous infection. Clin Geriatr Med.

[10] Kuijper E, Barbut F (2019). Clostridium and clostridioides. Manual of Clinical Microbiology.

[11] García-Jiménez A, Prim N, Crusi X, Benito N (2016). Septic arthritis due to clostridium ramosum. Semin Arthritis Rheum.

[12] Fauser DJ, Zuckerman JD (1988). Clostridial septic arthritis: case report and review of the literature. Arthritis Rheum.

[13] Lazzarini L, Conti E, Ditri L, Turi G, de Lalla F (2004). Clostridial orthopedic infections: case reports and review of the literature. J Chemother.

[14] Kurnutala LN, Ghatol D, Upadhyay A (2015). Clostridium sacroiliitis (gas gangrene) following sacroiliac joint injection--case report and review of the literature. Pain Physician.

[15] Vijayvargiya P, Garrigos ZE, Rodino KG, Razonable RR, Saleh OMA (2020). Clostridium paraputrificum septic arthritis and osteomyelitis of shoulder: a case report and review of literature. Anaerobe.

